# Robust Reverberation Suppression Method Based on Alternating Projections

**DOI:** 10.3390/s25030939

**Published:** 2025-02-04

**Authors:** Xiongwei Xiao, Feng Xu, Juan Yang

**Affiliations:** Ocean Acoustic Technology Laboratory, Institute of Acoustics, Chinese Academy of Sciences, Beijing 100190, China; xiaoxiongwei@mail.ioa.ac.cn (X.X.); xf@mail.ioa.ac.cn (F.X.)

**Keywords:** reverberation suppression, alternating projections, moving target detection, low rank and sparse matrix decomposition

## Abstract

By leveraging the high correlation between multi-ping echo data, low-rank and sparse decomposition methods are applied for reverberation suppression. Previous methods typically perform decomposition on the vectorized multi-ping echograph, which is obtained by stacking beamforming outputs from all directions in the same column. However, when the multi-ping correlation of beamforming outputs from different directions varies significantly due to the time-varying nature of the underwater acoustic channel, it becomes challenging to precisely capture the variations of the reverberation background. As a result, the performance of reverberation suppression is degraded. To alleviate this issue, we attempt to decompose the matrix formed by multi-ping beamforming outputs in different directions individually. The accelerated alternating projections method is used to estimate the steady reverberation for moving target detection. By exploiting the differences in spatio-temporal dimensions between moving targets and reverberation fluctuations, a weighted spatio-temporal density method with adaptive thresholding is used to further extract the target echoes. Field data were utilized to validate the effectiveness of the proposed method, and the experimental results demonstrated its superior robustness in an unstable reverberation-limited environment, maintaining an accurate estimation of steady reverberation.

## 1. Introduction

Active sonar plays a crucial role in protecting vital maritime facilities from underwater threats, such as divers and unmanned underwater vehicles [1,2]. One of the primary factors affecting the detection performance of active sonar is reverberation; it is the sum of scattered waves generated by numerous irregular scatters in the ocean at a certain time [3]. Reverberation in shallow water exhibits similar characteristics to target echoes in both frequency and time domains, making it challenging to distinguish targets and resulting in a high false alarm probability for active sonar systems [4]. Therefore, this paper focuses on improving the detection performance of active sonar in a reverberant environment.

Some research models reverberations from the perspective of their statistical characteristics. When the effective number of scatters within the resolution cell of a sonar is large enough, the reverberation statistics are expected to exhibit a Rayleigh probability distribution function (PDF) [5]. However, the matched-filter envelope in shallow water has shown a non-Rayleigh nature [6]. To fit the observed amplitude distributions, various statistics models have been proposed, such as log-normal [7], Weibull [8], and generalized Pareto [9] distributions. More accurate mixture models, including K–K distribution [10] and Poisson–Rayleigh distribution [11], are investigated. Nevertheless, due to the difficulty in parameter estimation and limited generality, a certain statistical model based on specific physical assumptions cannot be applied to all scenarios [12]. By utilizing the Doppler shift characteristics of moving targets, reverberation can be reduced in the Doppler domain [13,14]. However, such Doppler-based methods may be invalid for targets with lower speeds or in cases of high-level background fluctuations [15]. Based on the focusing characteristics of linear frequency-modulated (LFM) signals in the fractional Fourier domain, several approaches have been applied to detect moving targets in reverberant environments [16,17]. However, the effectiveness of these methods might be degraded in scenarios with a low signal-to-reverberation ratio (SRR) [15].

**Figure 1:**
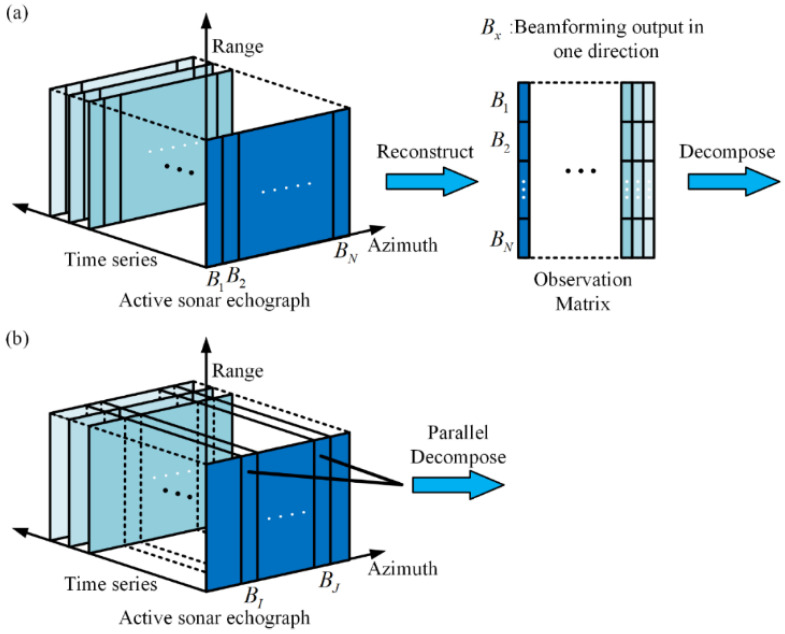
(**a**) Flowchart of previous reverberation suppression method. (**b**) Flowchart of the proposed reverberation suppression method.

In the context of underwater surveillance of maritime areas, active sonar is typically deployed in a fixed position, resulting in highly correlated echo signals over consecutive pulse cycles [4]. These returns are generally displayed as echographs, showing echo intensity across various ranges and bearings [14]. By stacking multiple vectorized echographs into an observation matrix (as shown in Figure 1a), methods based on low-rank and sparsity matrix decomposition can decompose it into dynamic and steady component [18]. The dynamic component, consisting of echoes from moving targets and oceanic fluctuations (e.g., surface waves) [15], is treated as a sparse matrix, while the steady component that arises from the stationary state of the ocean, like the seabed [19], is treated as a low-rank matrix. Rooted in this idea, fruitful methods have been proposed for reverberation suppression, including the accelerated proximal gradient (APG) method [20], the dynamic mode decomposition method [21], and the alternate direction multiplier method (ADMM) [15,22].

The aforementioned methods perform well when the underwater environment is relatively stable, as this ensures that the correlation of multi-ping beamforming outputs across different directions remains highly consistent. However, due to the time-varying characteristics of the underwater acoustic channel, these correlations can vary greatly. The fluctuation in sound propagation in different directions makes it challenging to accurately estimate the low-rank background, leading to the obscuration of weak target signal; thus, the above-mentioned method may lead to reverberation suppression performance degradation.

To address this issue, we propose a more robust method for detecting small moving targets in a reverberation-limited environment. Specifically, we try to perform low-rank and sparsity decomposition on the matrix formed by multi-ping beamforming outputs in particular directions separately (as shown in Figure 1b). By utilizing parallel computing, it is possible to process beamforming outputs from multiple directions simultaneously, thereby avoiding any substantial increase in computational time.

In this paper, we achieve reverberation suppression in two steps. The first step is to suppress the steady component of reverberation. For the actual water environment, estimating the steady component in nonlinear space may be more appropriate, as some research has indicated. Xiang et al. [23] employed a robust autoencoder method to project the echo data into a low-dimensional space, achieving a nonlinear estimation of the steady component of reverberation. Zhu et al. [24] utilized the algorithm of the Grassmann manifold to obtain a low-rank matrix, thereby reducing computational time while maintaining the performance of reverberation suppression. We try to estimate the steady reverberation in the Riemann manifold using the accelerated alternating projections method [25], and the dynamic component is obtained via a hard thresholding operator. It is worth emphasizing that we process the multi-ping beamforming output in each direction in parallel and aggregate the results to obtain the complete dynamic components. After eliminating the steady component of reverberation, the target echo signals can be detected in the dynamic component by setting a hard threshold in most cases. However, the fluctuation characteristic of the underwater acoustic environment causes the reverberation fluctuations and target echoes to exhibit comparable magnitudes in the dynamic component of some pings [15]. Thus, the second step is to further extract target echoes from dynamic components. Noting that the target signal and reverberation fluctuations show differences in weighted spatio-temporal density (WSTD) [26] features, a WSTD threshold can be set to distinguish the reverberation fluctuations and the target echoes. However, the determination of the WSTD threshold poses challenges. In this paper, we select the threshold based on the WSTD distribution characteristics of reverberation fluctuations in each ping, enabling a more effective extraction of the target echoes.

The remainder of this paper is organized as follows. In Section 2, we analyze the correlation of beamforming outputs in different directions using correlation coefficients. Section 3 introduces a method for steady reverberation suppression based on accelerated alternating projections, followed by the suppression of reverberation fluctuations using the WSTD method. We also introduce two evaluation metrics for reverberation reduction performance: bias of the Integral SideLobe Ratio and the sparse coefficient. In Section 4, we analyze the performance of the proposed method using field data and compare it with other methods. Section 5 summarizes the conclusions of this study.

## 2. Correlation Analysis of Multi-Ping Beamforming Output

In the case where the active sonar is positioned in a fixed location, it periodically emits pulse signals and receives the echoes. One ping of these echoes can be represented using a bearing-range spatial spectrum matrix $X_{i}\in R^{N_{r}\times N_{\theta }}(i=1,2,\ldots N)$, where $N_{r}$ and $N_{\theta }$ are the dimensions in the range and bearing, respectively. We then define $x_{i,j}\in {\mathbb{R}}^{N_{r}\times 1}(j=1,2,\ldots N_{\theta })$ as the beamforming and match-filtered output of a direction within $X_{i}$. We then reorganize the matrix $X_{i}$ as a column vector $m_{i}\in {\mathbb{R}}^{N_{r}N_{\theta }\times 1}$, which is

$$m_{i}=\left[\begin{array}{l} x_{i,1}\\ x_{i,2}\\ \;\vdots \\ x_{i,N_{\theta }} \end{array}\right]$$

N pings of $m_{i}$ form an observation matrix $M\in {\mathbb{R}}^{N_{r}N_{\theta }\times N}$ as follows:

$$\begin{array}{l} M=[m_{1},m_{2},\ldots m_{N}]\\ \;=\;\left[\begin{array}{ccc} \begin{array}{c} x_{1,1}\\ x_{1,2}\\ \;\vdots \\ x_{1,N_{\theta }} \end{array} & \begin{array}{l} x_{2,1}\\ x_{2,2}\\ \;\vdots \\ x_{2,N_{\theta }} \end{array} & \begin{array}{l} \begin{array}{cc} \cdots & x_{N,1} \end{array}\\ \begin{array}{cc} \cdots & x_{N,2} \end{array}\\ \begin{array}{cc} & \vdots \end{array}\\ \begin{array}{cc} \cdots & x_{N,N_{\theta }} \end{array} \end{array} \end{array}\right] \end{array}$$

Previous methods typically perform matrix low-rank and sparse decomposition on such observation matrices to achieve steady reverberation suppression. When the moving targets enter the monitoring area of the active sonar, M can be treated as a sum of a low-rank matrix L and sparse matrix S as follows:

(1) M=L+S

where L contains the steady component of reverberation and S contains the moving target echoes and reverberation fluctuations. In the scenario where the underwater acoustic environment remains stable over time, the column vectors of M exhibit strong correlations. The column correlations between the submatrices of $M_{k}=[x_{1,k},x_{2,k},\ldots ,x_{N,k}],(k=1,2,\ldots N_{\theta })$ are highly congruent; thus, the previous methods show good performance. However, due to the time-varying characteristics of the actual underwater acoustic channel, the correlation of beamforming and match-filtered outputs in different directions may vary greatly. That is to say, the column correlations between $M_{k_{1}}=[x_{1,k_{1}},x_{2,k_{1}},\ldots ,x_{N,k_{1}}]$ and $M_{k_{2}}=[x_{1,k_{2}},x_{2,k_{2}},\ldots ,x_{N,k_{2}}]$ may differ significantly, where $k_{1}$ and $k_{2}$ represent the index of two directions. Therefore, if M is directly subjected to low-rank sparse decomposition, the inconsistent column correlation of its submatrices $M_{k}$ will result in an inaccurate estimation of the reverberation background.

**Figure 2:**
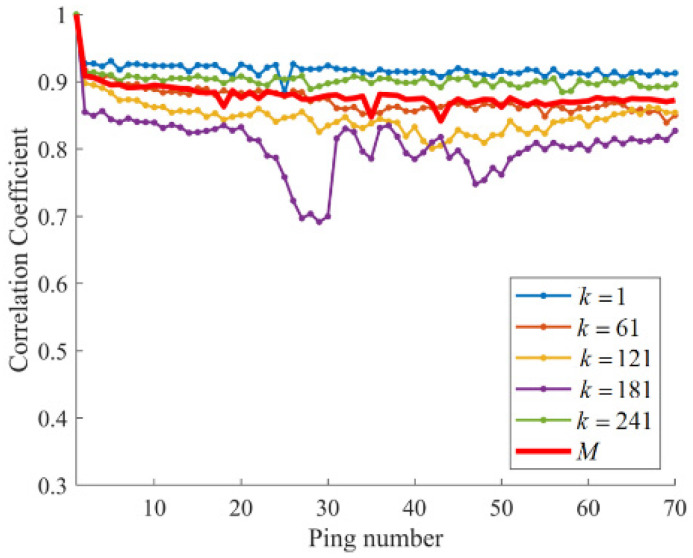
The correlation coefficient analysis of the received data.

To quantitatively describe the column correlations of M and $M_{k}$, the correlation coefficient is employed as the evaluation criterion. This coefficient measures the strength of the relationship between two vectors and is defined as follows:

(2) $$R(v_{1},v_{2})=\frac{\sum_{i=1}^{n}\left(v_{1,i}-{\bar{v}}_{1})(v_{2,i}-{\bar{v}}_{2}\right)}{\sqrt{\sum_{i=1}^{n}{\left(v_{1,i}-{\bar{v}}_{1}\right)}^{2}\sum_{j=1}^{n}{\left(v_{2,i}-{\bar{v}}_{2}\right)}^{2}}}$$

where $\bar{\left(\cdot \right)}$ is the mean of vector elements. The larger correlation coefficient indicates a stronger correlation between two vectors. We analyzed a segment of shallow sea reverberation data collected during a sea trial experiment. The details of the data collection are elaborated in Section 4.1. In this context, $N_{\theta }=256$, corresponding to 360°. The first column in each matrix is utilized as the reference vector. Specifically, $m_{1}$ and $x_{1,k}$ are designated as reference vectors of M and $M_{k}$. The indices chosen are $k=\left\{1,61,121,181,241\right\}$. We calculate the correlation coefficient between the reference vector and other columns. The results are depicted in Figure 2. As can be seen in Figure 2, there is a relatively high correlation among the columns of M, which gradually decreases over time, with significant declines observed at pings 18, 35, and 43. This indicates that these pings exhibit considerable fluctuations in strength compared to the other pings. The column correlations between $M_{k}$ vary and may not follow the same trend as that of M. The column correlations of $M_{1}$ and $M_{241}$ essentially remain at a consistently close and stable level, indicating that the acoustic channel in these directions remains relatively stable, resulting in relatively consistent intensity. In contrast, the column correlations of $M_{121}$ and $M_{181}$ exhibit significant variations, with sudden changes occurring in some pings. This might imply that there are strong fluctuations in the acoustic channel in these directions. Only $M_{61}$ exhibits column correlations that are relatively similar to those of M. In summary, the intensity fluctuations of specific submatrices cannot be precisely reflected by the column correlations of $M_{n}$, as the column correlations of other submatrices may dominate or mask the impact of M, thereby diminishing its contribution to the overall correlation. This could lead to inaccuracies in reverberation background estimation, which may, in turn, cause the target echoes in the dynamic component to be weak or even undetectable. Hence, we propose a more robust method to achieve steady reverberation suppression. We perform low-rank and sparse matrix decomposition on $M_{k}\in {\mathbb{R}}^{N_{r}\times N},(k=1,2,\ldots N_{\theta })$ separately.

## 3. Methodology of Reverberation Suppression

### 3.1. Problem Formulation

Suppose we have multi-ping data $𝒳=[X_{1},X_{2},\ldots X_{N}]\in {\mathbb{R}}^{N_{r}\times N_{\theta }\times N}$; the matrix formed by multi-ping beamforming output in a particular direction is a slice of 𝒳 along time dimensions $M_{k}\in {\mathbb{R}}^{N_{r}\times N},(k=1,2,\ldots N_{\theta })$. According to the analysis in Section 2, $M_{k}$ can be modeled as the sum of the steady component and dynamic component as follows:

(3) $$M_{k}=L_{k}+S_{k}$$

where $L_{k}\in {\mathbb{R}}^{N_{r}\times N}$ contains the coherent part of $M_{k}$ and $S_{k}$ contains the incoherent part in $M_{k}$. Clearly, $L_{k}$ is a low-rank matrix representing the steady reverberation that originates from the static state of the ocean, such as the sea bottom. $S_{k}$ is a sparse matrix that cpatures both targets echoes and reverberation fluctuations in the presence of target , and only reverberation fluctuations in the absence of targets. We consider the scenario in which the targets are present.

### 3.2. Reducing Steady Reverberation Using AccAltProj

Reverberation suppression aims to enhance the detection of target echo signals in a reverberation-limited environment. Given that the target echoes are present within the dynamic component, it is essential to extract the sparse matrix $S_{k}$.

In this paper, we try to achieve the decomposition of Equation (3) by a non-convex algorithm. The objective function of Equation (3) can be formulated as

(4) $$\underset{L_{k},S_{k}\in {\mathbb{R}}^{N_{r}\times N}}{\min}{\left\|M_{k}-L_{k}-S_{k}\right\|}_{F}\;s.t.\;\mathrm{rank}(L_{k})\leq r\;\mathrm{and}\;{\left\|S_{k}\right\|}_{0}\leq \left|\Omega \right|,$$

where r denotes the rank of the underlying low-rank matrix, Ω denotes the support set of the underlying sparse matrix, and ${\left\|\;\cdot \;\right\|}_{0}$ denotes the $l_{0}$ norm of a matrix. The solution to Equation (4) can be achieved through the method of alternating projections [25]. In each iteration, $L_{k}$ could be obtained by projecting $M_{k}-S_{k}$ onto the space of rank-r matrices, denoted ${ℳ}_{r}$, and $S_{k}$ could be obtained by projecting $M_{k}\;-\;L_{k}$ into the space of sparse matrices, denoted Ƶ. The algorithm consists of two phases: initialization and projections on to ${ℳ}_{r}$ and Ƶ alternatively.

Let $\left(L_{k}^{\left(n\right)},S_{k}^{\left(n\right)}\right)$ denote the current estimations. At the (n+1)-th iteration, the left and right singular vectors define an $(N_{r}N-r)r$-dimensional subspace [25,27].

(5) $$T_{k}^{\left(n\right)}=\left\{U_{k}^{\left(n\right)}A^{T}+BV_{k}^{(n)T}\left|A,B\in {\mathbb{R}}^{N_{r}\times r}\right.\right\}$$

where $L_{k}^{\left(n\right)}=U_{k}^{\left(n\right)}\Sigma_{k}^{\left(n\right)}V_{k}^{(n)T}$ is the SVD of $L_{k}^{\left(n\right)}$. The projections of a given matrix $E\in {\mathbb{R}}^{N_{r}\times N}$ onto the subspace $T_{k}^{\left(n\right)}$ are given by

(6) $${𝒫}_{T_{k}^{\left(n\right)}}E=U_{k}^{\left(n\right)}U_{k}^{(n)T}E+EV_{k}^{\left(n\right)}V_{k}^{(n)T}-U_{k}^{\left(n\right)}U_{k}^{(n)T}EV_{k}^{\left(n\right)}V_{k}^{(n)T}$$

Then, this intermediate matrix can be projected onto the rank-r matrix manifold $M_{r}$ by truncated SVD, which is

(7) $$L_{k}^{\left(n+1\right)}={𝓗}_{r}({𝒫}_{T_{k}^{\left(n\right)}}(M_{k}-S_{k}^{\left(n\right)}))$$

where ${𝓗}_{r}$ computes the best rank-r approximation of the given matrix K

(8) $${𝓗}_{r}(K)=Q\Lambda_{r}P^{T}$$

where $K=Q\Lambda P^{T}$ is its SVD and ${\left[\Lambda_{r}\right]}_{ii}=\left\{\begin{array}{l} \Lambda_{ii}\;i\leq r\\ 0\;\mathrm{otherwise}. \end{array}\right.$

Utilizing QR decomposition offers a more efficient and accurate approach to estimate $L_{k}^{\left(n+1\right)}$ [25]. Let $(I-U_{k}^{\left(n\right)}U_{k}^{(n)T})(M_{k}-S_{k}^{\left(n\right)})V_{k}=Q_{1}R_{1}$ and $(I-V_{k}^{\left(n\right)}V_{k}^{(n)T})(M_{k}-S_{k}^{\left(n\right)})U_{k}^{\left(n\right)}=Q_{2}R_{2}$ be the QR decompositions of $(I-U_{k}^{\left(n\right)}U_{k}^{(n)T})(M_{k}-S_{k}^{\left(n\right)})V_{k}$ and $(I-V_{k}^{\left(n\right)}V_{k}^{(n)T})(M_{k}-S_{k}^{\left(n\right)})U_{k}^{\left(n\right)}$, respectively. Then, the projections of $M_{k}-S_{k}^{\left(n\right)}$ onto the tangent space of ${ℳ}_{r}$ could be calculated as follows [25]:

(9) $$\begin{array}{l} {𝒫}_{T_{k}^{\left(n\right)}}(M_{k}\;-\;S_{k}^{\left(n\right)})\;=\;U_{k}^{\left(n\right)}U_{k}^{(n)T}(M_{k}-S_{k}^{\left(n\right)})+(M_{k}-S_{k}^{\left(n\right)})V_{k}^{(n)T}V_{k}^{(n)T}-U_{k}^{\left(n\right)}U_{k}^{(n)T}(M_{k}-S_{k}^{\left(n\right)})V_{k}^{\left(n\right)}V_{k}^{(n)T}\\ \;=U_{k}^{\left(n\right)}U_{k}^{(n)T}(M_{k}-S_{k}^{\left(n\right)})(I-V_{k}^{\left(n\right)}V_{k}^{(n)T})+(I-U_{k}^{\left(n\right)}U_{k}^{(n)T})(M_{k}-S_{k}^{\left(n\right)})V_{k}^{\left(n\right)}V_{k}^{(n)T}\\ \;+U_{k}^{\left(n\right)}U_{k}^{(n)T}(M_{k}-S_{k}^{\left(n\right)})V_{k}^{\left(n\right)}V_{k}^{(n)T}\\ \;=U_{k}^{\left(n\right)}R_{2}^{T}Q_{2}^{T}+Q_{1}R_{1}V_{k}^{n}+U_{k}^{\left(n\right)}U_{k}^{(n)T}(M_{k}-S_{k}^{\left(n\right)})V_{k}^{(n)T}V_{k}^{(n)T}\\ \;=\left[U_{k}^{\left(n\right)}\;Q_{1}\right]\left[\begin{array}{cc} U_{k}^{(n)T}(M_{k}-S_{k}^{\left(n\right)})V & R_{2}^{T}\\ R_{1} & \;0\; \end{array}\right]\left[\begin{array}{l} V_{k}^{(n)T}\\ Q_{2}^{T} \end{array}\right]\\ \;=\left[U_{k}^{n}\;Q_{1}\right]E_{k}^{n}\left[\begin{array}{l} V_{k}^{nT}\\ Q_{2}^{T} \end{array}\right] \end{array}$$

where the fourth line follows from the fact $U_{k}^{(n)T}Q_{1}=V_{k}^{(n)T}Q_{2}=0$. Let $E_{k}^{\left(n\right)}=U_{E_{k}^{\left(n\right)}}\Sigma_{E_{k}^{\left(n\right)}}V_{E_{k}^{\left(n\right)}}^{T}$ be the SVD of $E_{k}^{\left(n\right)}$. Then, the SVD of ${𝒫}_{T_{k}^{\left(n\right)}}(M_{k}-S_{k}^{\left(n\right)})=U_{k}^{\left(n\right)}\Sigma_{k}^{\left(n\right)}V_{k}^{(n)T}$ can be computed by

$$U_{k}^{\left(n+1\right)}=[U_{k}^{\left(n\right)}\;\;\;\;Q_{1}]U_{E_{k}^{\left(n\right)}}\;\Sigma_{k}^{\left(n+1\right)}=\Sigma_{E_{k}^{\left(n\right)}}$$

and

$$V_{k}^{\left(n+1\right)}=\left[V_{k}^{\left(n\right)}\;\;\;\;Q_{2}\right]V_{E_{k}^{\left(n\right)}}$$

The computational complexity is $4n^{2}r+n^{2}+O(nr^{2}+r^{3})$ flops. As $L_{k}^{\left(n+1\right)}$ is obtained, we further update the sparse matrix using the hard thresholding operator [25]

(10) $$S_{k}^{\left(n+1\right)}={𝒯}_{\xi_{n+1}}(M_{k}-L_{k}^{\left(n+1\right)})$$

where the thresholding operator ${𝒯}_{\xi_{n+1}}$ is defined for any matrix K as follows:

(11) $${\left[{𝒯}_{\xi_{n+1}}K\right]}_{ij}=\left\{\begin{array}{l} {\left[K\right]}_{ij}\;\left|{\left[K\right]}_{ij}\right|>\xi_{n+1}\\ 0\;\mathrm{otherwise} \end{array}\right.$$

the thresholding value of $\xi_{n+1}$ is chosen as follows [25]:

(12) $$\xi_{n+1}=\beta (\sigma_{r+1}({𝒫}_{T_{k}^{n}}(M_{k}-S_{k}^{\left(n\right)})))+\gamma^{n+1}\sigma_{1}(({𝒫}_{T_{k}^{n}}(M_{k}-S_{k}^{\left(n\right)})))$$

where the β>0 is a tunning parameter, γ is the convergence rate parameter and set to be 0.7 in this experiment, and σ is the singular values of ${𝒫}_{T_{k}^{\left(n\right)}}(M_{k}-S_{k}^{\left(n\right)})$, which have been obtained when computing $L_{k}^{\left(n+1\right)}$. We follow the initialization proposed in [25].

Based on the aforementioned methods for estimating $L_{k}$ and $S_{k}$, we then introduce the reverberation suppression method proposed in this paper. Given the original data $𝒳\in {\mathbb{R}}^{N_{r}\times N_{\theta }\times N}$, the k-th slice of 𝒳 along the third dimension is $M_{k}=𝒳(:,k,:)$. The decomposition of 𝒳 is described in Algorithm 1.

**Algorithm 1:** Steady reverberation reduction based on AccAltProjInput: Echographs Tensor $𝒳\in {\mathbb{R}}^{N_{r}\times N_{\theta }\times N}$Initilize: $𝒮\in {\mathbb{R}}^{N_{r}\times N_{\theta }\times N},𝒮=0$Parameter: r: target rank; ε: target precision level; β: thresholding parameter; γ: target converge rate;   1: **parfor** $k=1:N_{\theta }$
**do**          //parallel computing   2: **Initialize** $L_{k}$ and $S_{k}$ using initialization proposed in [25].   3: n=0   4: **while** ${{\left\|M_{k}-L_{k}-S_{k}\right\|}_{F}/\left\|M_{k}\right\|}_{F}\geq \varepsilon$
**do**   5: Estimate the steady component of reverberation $L_{k}^{\left(n+1\right)}$ using Equation (7)   6: Updata the hard thresholding value $\xi_{n+1}$ using Equation (12)   7: Estimate the dynamic component $S_{k}^{\left(n+1\right)}$ using Equation (10)   8: n=n+1   9: **end while**   10: $𝒮(:,k,:)=S_{k}$   11: **end parfor****Output**: 𝒮

### 3.3. Reducing Reverberation Fluctuations Using WSTD Method with Adaptive Thresholding

The steady reverberation interference has been eliminated by Algorithm 1, and the dynamic component 𝒮 is obtained. However, it contains not only moving target echoes but also reverberation fluctuations. These randomly occurring interference signals are spatially discrete and of short duration. In contrast, moving target echoes tend to be spatially concentrated and exhibit strong temporal continuity. Furthermore, the signal intensity of moving targets is typically stronger than that of reverberation fluctuations. Building upon these characteristics, the WSTD method [26] is proposed to further suppress the reverberation fluctuations. The WSTD of moving targets is different from that of clutter. By setting an appropriate WSTD threshold, the interference caused by reverberation fluctuations can be filtered out.

For the i-th ping input data $X_{i}\in {\mathbb{R}}^{N_{r}\times N_{\theta }}$, the dynamic component is estimated as ${𝒮}^{\left(i\right)}\in {\mathbb{R}}^{N_{r}\times N_{\theta }}$. By applying a hard threshold $\zeta_{i}$ to the entries in ${𝒮}^{\left(i\right)}$, the locations of potential moving targets were estimated as follows:

(13) $${𝒮}_{m,n}^{\prime (i)}=\left\{\begin{array}{l} {𝒮}_{m,n}^{\left(i\right)},{𝒮}_{m,n}^{\left(i\right)}>\zeta_{i}\\ 0,{𝒮}_{m,n}^{\left(i\right)}<\zeta_{i} \end{array}\right.,m=1,\ldots ,N_{r},n=1,\ldots ,N_{\theta },i=1,\ldots ,N$$

The non-zero entries in matrix ${{𝒮}^{\prime }}^{\left(i\right)}$ signify the location of potential moving targets in i-th ping; otherwise, there are no moving targets. To better detect the target echoes from ${𝒮}^{\left(i\right)}$, the threshold $\zeta_{i}$ is determined under a false alarm probability ($p_{fa}$) of 0.5 × 10^−3^. Then, we calculate the WSTD feature of 𝒮. For each non-zero entry in ${𝒮}^{\prime }$, the spatio-temporal window is centered around it with size $\left(2D_{r},2D_{\theta },2D_{f}\right)$, where $D_{r}$ is the window length in range dimension, $D_{\theta }$ is the window length in bearing direction dimension, and $D_{f}$ is the length in time dimension. The spatio-temporal density is the number of elements within its the spatio-temporal window, denoted by ${𝒢}_{m,n}^{\left(i\right)}$. The weights are obtained by normalizing the signal intensity values in ${𝒮}^{\left(i\right)}$ into a range between 0 and 1 as follows:

(14) $${𝒲}^{\left(i\right)}=\frac{{𝒮}^{\left(i\right)}-\min({𝒮}^{\left(i\right)})}{\max({𝒮}^{\left(i\right)})-\min({𝒮}^{\left(i\right)})}\;\;\;\;\;i=1,\ldots ,N$$

Then, the weighted spatio-temporal density feature map of ${𝒮}^{\prime }$ can be obtained as follows:

(15) $$F^{\left(i\right)}={𝒲}^{\left(i\right)}⊗{𝒢}^{\left(i\right)}\;\;\;\;\;i=1,\ldots ,N$$

where ⊗ denotes the Hadamard product of two matrices. Each non-zero entry in matrix $F^{\left(i\right)}$ reflects the weighted spatio-temporal density of the corresponding entry in ${𝒮}^{\prime }$. The spatio-temporal density, weighted by normalized signal intensity, maximizes the distinction between moving target echoes and reverberation fluctuations. The WSTD values for reverberation fluctuations are typically smaller and more numerous, whereas the WSTD values for moving targets are generally larger and constitute only a small fraction. Consequently, the threshold $Th_{WSTD}^{\left(i\right)}$ can be determined by leveraging the distribution characteristics of WSTD within $F^{\left(i\right)}$. We first calculate the histogram distribution of $F^{\left(i\right)}$ by employing 20 bins to discretize the data. It is observed that clutter is predominantly concentrated within the bin with the highest counts, and the upper limit of this bin can be adopted as the threshold $Th_{WSTD}^{\left(i\right)}$.

The target echo component, with the majority of reverberation fluctuations suppressed, can be obtained as follows:

(16) $${𝒮}_{m,n}^{\prime \prime (i)}=\left\{\begin{array}{l} {{𝒮}^{\prime }}_{m,n}^{\left(i\right)},F_{m,n}^{\left(i\right)}>Th_{WSTD}^{\left(i\right)}\\ 0,F_{m,n}^{\left(i\right)}<Th_{WSTD}^{\left(i\right)} \end{array}\right.,m=1,\ldots ,N_{r},n=1,\ldots ,N_{\theta },i=1,\ldots ,N$$

### 3.4. Evaluation Metric

To quantitatively analyze the reverberation suppression performance of the proposed method, two evaluation metrics are introduced: bias of the Integral SideLobe Ratio and the sparse coefficient.

#### 3.4.1. Bias of the Integral SideLobe Ratio

The Integral SideLobe Ratio (ISLR) represents the ratio of energy between the main lobe and the side lobes. A higher ISLR value indicates superior detection performance [28]. The ISLR is defined as follows:

(17) $$ISLR=10\mathrm{lg}E_{m}/E_{s}$$

where $E_{m}$ and $E_{s}$ denotes the total energy of the main lobe and the total energy of the side lobe. Reverberation suppression performance can be quantitatively evaluated by calculating the bias in the ISLR before and after suppression. $B_{ISLR}$ is defined as follows:

(18) $$B_{ISLR}=ISLR_{after}-ISLR_{before}$$

$B_{ISLR}$ serves as an index to evaluate the reverberation suppression performance. A greater value of $B_{ISLR}$ signifies that a larger portion of the reverberation has been suppressed.

#### 3.4.2. Sparse Coefficient

In the context of reverberation suppression, the dynamic component 𝒮 is the portion of interest as it contains the target echo signal. Therefore, the mathematical properties of the dynamic component 𝒮 largely determine the degree of suppression to reverberation, which could be evaluated with the sparse coefficient (SC) [15]. The dynamic component is expected to have high sparsity. A greater SC implies a larger number of zero entries or entries with minor values in 𝒮. In this paper, we evaluate the SC of matrix ${𝒮}^{\left(i\right)}$, which is defined as follows:

(19) $$SC_{i}=1-\frac{N_{{𝒮}^{\left(i\right)}}\left(\mu \right)}{N_{r}N_{\theta }}$$

where $N_{{𝒮}^{\left(i\right)}}\left(\mu \right)$ is the total number of absolute values of entries in ${𝒮}^{\left(i\right)}$ greater than μ. The $SC_{i}$ primarily originates from the reverberation fluctuations; a greater value of $SC_{i}$ indicates a weaker intensity of reverberation fluctuations.

## 4. Experiments

### 4.1. Field Data Collection and Preprocessing

**Figure 3:**
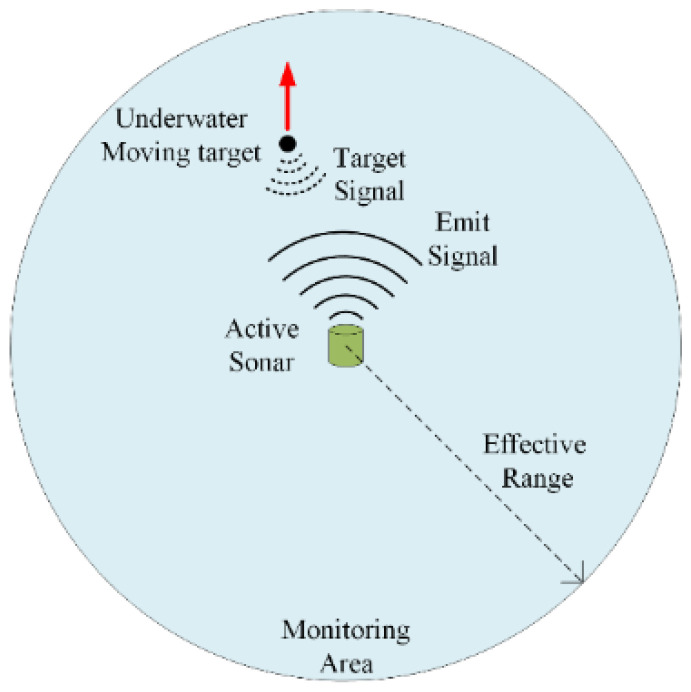
Schematic of the experiment. The red arrow indicates the approximate direction of movement of the target.

To validate the effectiveness of the proposed method for reverberation reduction, a field experiment was conducted in a certain maritime area in Sanya, Hainan Province, China. The water depth is approximately 8.7 m, and the sound speed is about 1531 m/s. A high-frequency active sonar is deployed in a fixed position, periodically emitting pulse signals into the water and receiving echoes, with the signal waveform being LFM. The sonar detection range is 500 m, with a field of view of 360°. The underwater small target moves at a depth of approximately 5 m. A GPS device was placed directly above the underwater small target to record its trajectory. Figure 3 depicts the experimental schematic. A total of 70 pings of bearing-range spatial spectrum data were extracted to validate the performance of the proposed method. The experiment was carried out in shallow water where reverberation dominated over ambient noise; hence, the impact of ambient noise was not taken into consideration.

### 4.2. Experimental Results and Discussion

In this section, the performance of the proposed method for reverberation suppression is analyzed using field data. Section 4.2.1 illustrates the method’s effectiveness in suppressing the steady component of reverberation and compares it with the other method. Section 4.2.2 demonstrates the performance improvements achieved after suppressing reverberation fluctuations.

#### 4.2.1. Steady Reverberation Suppression

**Figure 4:**
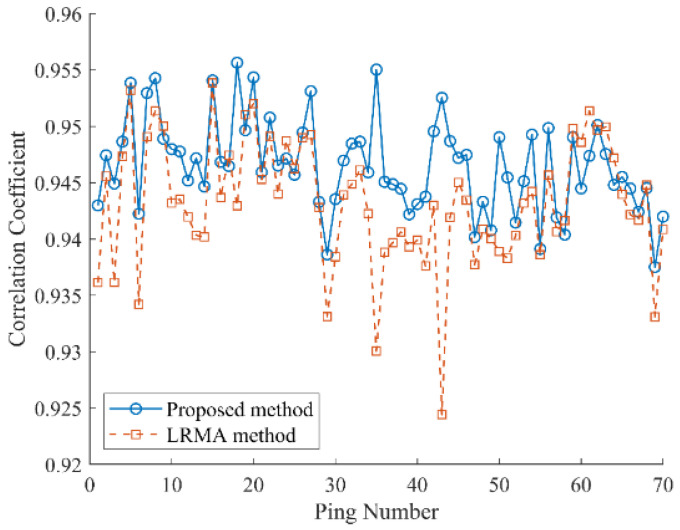
Correlation coefficient between the original data and the steady component.

To validate the effectiveness of the proposed method, we compare it with the low-rank matrix approximation (LRMA) [29] method, which performs decomposition on the observation matrix shown in Figure 1a, and the final results are obtained by inverse operation. In this experiment, the target rank is set to 2 and the target precision level ε is set to 10^−6^. The moving target signal typically occupies only a small portion of the original echograph, meaning the estimated steady reverberation should have a high correlation with the original data. To quantitatively assess the accuracy of the estimated steady reverberation background, the correlation coefficient between the steady reverberation and the original data is calculated. These coefficients, determined using Equation (2), are shown in Figure 4. As illustrated in Figure 4, in pings where the reverberation background fluctuates significantly, such as pings 18, 35, and 43, the proposed method’s estimated steady reverberation maintains a high correlation with the original data. In contrast, the correlation of the reverberation background estimated by the LRMA method decreases in these pings. To further illustrate the impact of accurate reverberation background estimation on the extraction of moving target signals, pings 35 and 43 were subsequently analyzed.

**Figure 5:**
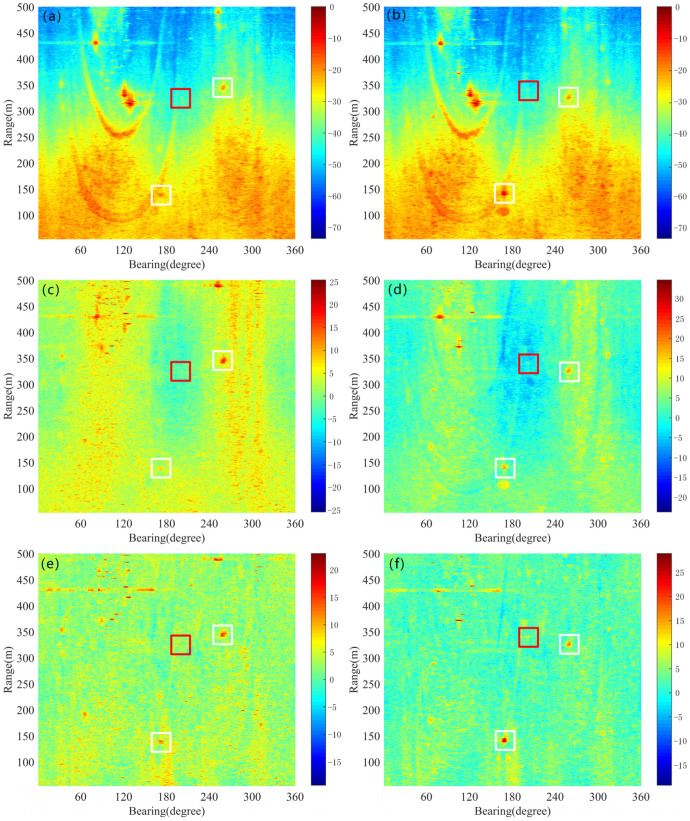
Reverberation suppression for pings 35 and 43 by the proposed method and the LRMA method; the target is shown in the red rectangle and the ships are shown in white rectangles. (**a**) Original data of ping 35. (**b**) Original data of ping 43. (**c**) Dynamic component of ping 35 by the LRMA method. (**d**) Dynamic component of ping 43 by the LRMA method. (**e**) Dynamic component of ping 35 by the proposed method. (**f**) Dynamic component of ping 43 by the proposed method.

The original data for pings $X_{35}$ and $X_{43}$ are presented in Figure 5a,b. In these figures, the underwater small target is marked with a red rectangular box, while the ships are indicated by white rectangular boxes. The target echo intensities in both pings are notably weak and are obscured by strong surrounding reverberation, making the targets nearly invisible. The dynamic components extracted using the LRMA method are shown in Figure 5c,d, where the target intensities are 4.11 dB and 5.86 dB, respectively. Although most of the steady reverberation has been eliminated, reverberation fluctuations still dominate, making the targets indistinguishable. In contrast, the dynamic components estimated by the proposed method, displayed in Figure 5e,f, have target intensities of 11.44 dB and 15.8 dB, respectively. Compared to the LRMA method, the proposed method extracts stronger target signals with fewer reverberation fluctuations. This demonstrates that accurate estimation of steady reverberation not only enhances target signal strength within the dynamic components but also reduces reverberation fluctuations, thereby making it easier to identify the targets’ locations.

**Figure 6:**
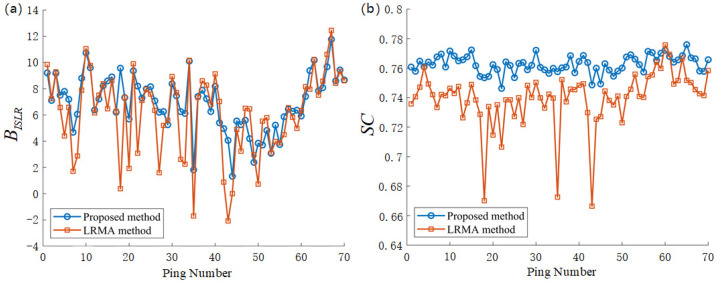
(**a**) $B_{ISLR}$ of all pings of the dynamic component by the LRMA method and the proposed method. (**b**) SC of all pings of the dynamic component by the LRMA method and the proposed method.

To quantitatively evaluate the performance of the proposed method on the field data, we calculate the $B_{ISLR}$ of the LRMA method and the proposed method. If the $B_{ISLR}$ is greater than zero, it signifies effective reverberation suppression; conversely, if it is less than zero, the suppression effect is inadequate. As presented in Figure 6a, the $B_{ISLR}$ values of the proposed method are mostly higher than those of the LRMA method. For ping 35, ping 43, and ping 44, the $B_{ISLR}$ values of the LRMA method are less than zero, and they are −1.44, −2.43, and −0.22, respectively, while the values for the proposed method are 1.81, 4.06, and 1.33. In these pings, the correlation coefficient with the reference vector rapidly decreases due to significant variations in the reverberation background. However, the LRMA method struggles to capture these changes, resulting in a lower $B_{ISLR}$. In contrast, the proposed method demonstrates robust performance under these conditions. The average $B_{ISLR}$ of the proposed method is 6.92, compared to 6.05 for the LRMA method, demonstrating the superiority of the proposed method. Let ζ in Equation (13) be set to 2; the SC values across 70 pings obtained by the proposed method and the LRMA method are presented in Figure 6b. It can be observed that the SC values for nearly all pings using the proposed method surpass those achieved by the LRMA method, particularly at pings 18, 35, and 43. The mean SC for the proposed method is 0.76, while that for the LRMA method is 0.73. Compared to the LRMA method, the SC values of the proposed method increased by a factor of 1.04. This suggests that the dynamic components obtained by the proposed method exhibit a superior reduction in reverberation fluctuations.

#### 4.2.2. Reverberation Fluctuation Suppression

**Figure 7:**
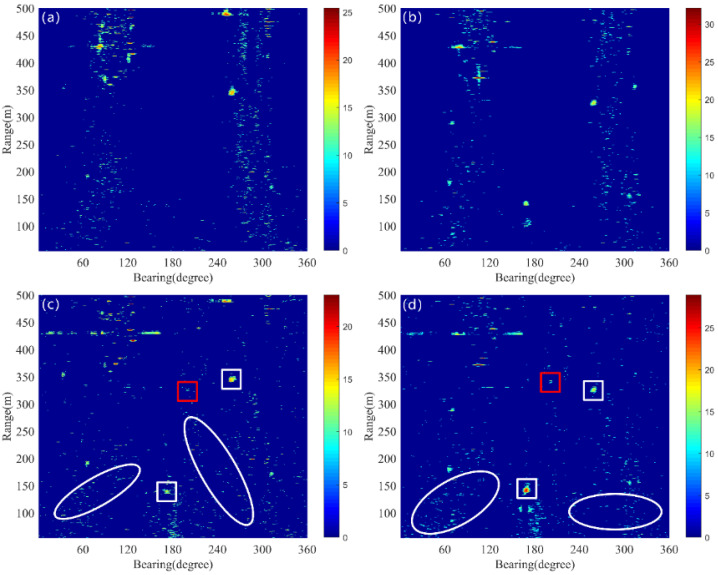
The red rectangle represents the underwater target, the white rectangle denotes the ship, and the white ellipse signifies the fluctuation of reverberation. (**a**) Thresholding result of ping 35 by the LRMA method. (**b**) Thresholding result of ping 43 by the LRMA method. (**c**) Thresholding result of ping 35 by the proposed method. (**d**) Thresholding result of ping 43 by the proposed method.

To better detect the target echo signal in a specific ping of dynamic component, the detecting threshold is determined under a $p_{fa}$ of 5 × 10^−3^. For this probability of a false alarm, the $\zeta_{35}$ and $\zeta_{43}$ are 10.87 dB and 11.28 for the LRMA method and 9.1 dB and 9.59 for the proposed method, respectively. As illustrated in Figure 7a, the LRMA method fails to detect target echoes, including those from underwater small targets and ships. Similarly, target echoes are invisible in Figure 7b. The detection results of the proposed method for the 35th and 43rd pings are shown in Figure 7c,d. One can observe that the echo of small targets becomes visible (as indicated by the red rectangular boxes), as well as ship targets (as indicated by the white rectangular boxes). However, there is significant interference raised from reverberation fluctuations in these two pings (as indicated by the white ellipse), which affects the identification of target echoes.

**Figure 8:**
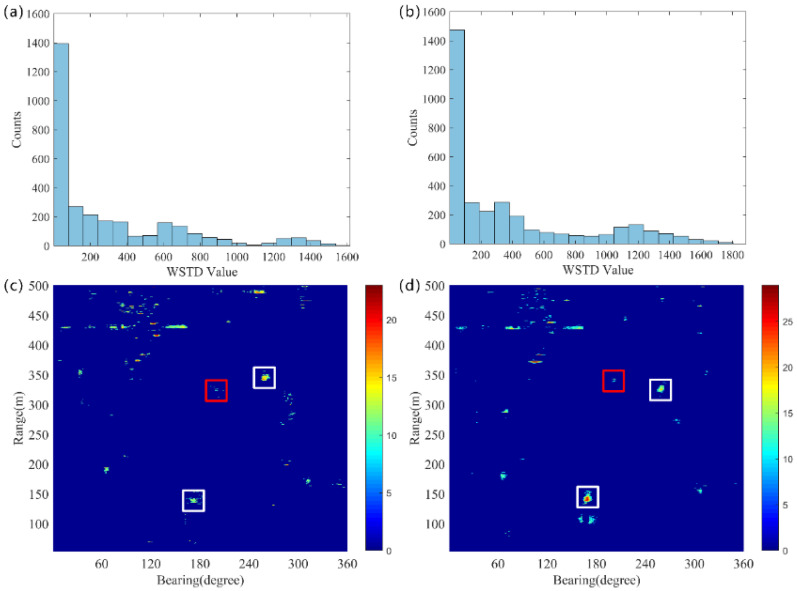
The red rectangle represents the underwater target, the white rectangle denotes the ship. (**a**) The histogram of the WSTD distribution for 35 ping. (**b**) The histogram of the WSTD distribution for 43 ping. (**c**) The target echoes extracted by the WSTD method for ping 35. (**d**) The target echoes extracted by the WSTD method for ping 43.

To further reduce the reverberation fluctuations, we calculate the WSTD feature of 𝒮. The distribution of WSTD for pings 35 and 43 is shown in Figure 5a,b. It can be seen that the WSTD of the reverberation fluctuations is concentrated between 0 and 100, while the WSTD of the target is usually greater than 100. Therefore, it is feasible to determine the threshold based on the distribution characteristics of the WSTD. We take the upper limit of the bins with the highest counts as the threshold, and the target echoes are further extracted through the WSTD method. The WSTD thresholds calculated for pings 35 and 43 are 81 and 95, respectively. As shown in Figure 8c,d, it is evident that the clutter interference caused by reverberation fluctuations has been effectively suppressed without reducing the intensity of the target echoes, allowing clear visibility of the target.

**Figure 9:**
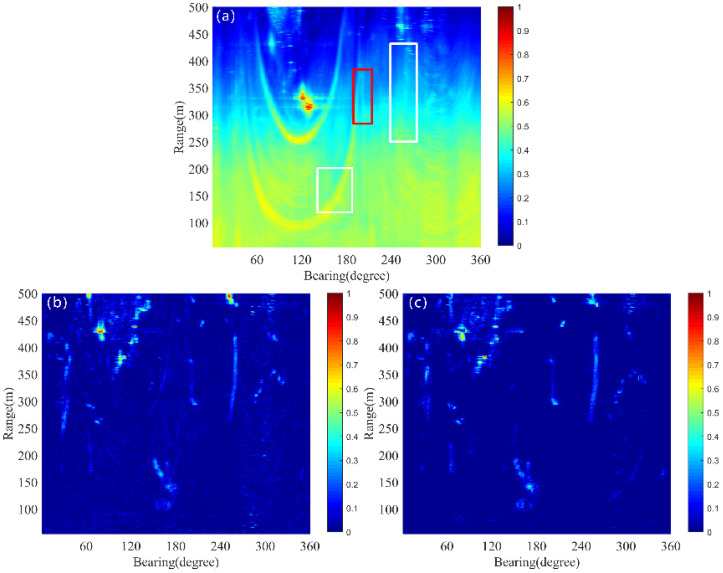
The reverberation reduction to field data of all pings by the LRMA method and the proposed method; the target trajectory is shown in a red rectangle and the ship trajectories are shown in white rectangles. (**a**) The original data. (**b**) The LRMA method. (**c**) The proposed method.

Overlaying the detection results from each ping produces a trajectory map that shows the trajectory of the moving target. The trajectories of underwater small targets are highlighted with red rectangles, while the trajectories of ships are marked with white rectangles. As shown in Figure 9a, the target echo signals are submerged in background interference, making it impossible to identify the target trajectory in the presence of severe reverberation. In Figure 9b, the result of the LRMA method is displayed; although the target trajectory becomes discernible, it shows discontinuities, indicating that the target is undetectable in certain pings. The reverberation is reduced to some extent, but considerable reverberation fluctuations still persist. Figure 9c shows the results obtained by the proposed method, where both steady reverberation and reverberation fluctuations are effectively suppressed, allowing a clear visibility of the target trajectory.

## 5. Discussion

In this experiment, the computer used for calculations was equipped with a Core i7-11800 h CPU and 32 GB of RAM. To evaluate the computational performance of the proposed method, multiple experiments were conducted, and the average value was taken as the final result. The LRMA method took 12.24 s to decompose 70 pings of original data. The proposed method took 0.53 s to compute the multi-ping beamforming output for a single direction and 17.85 s for all directions using parallel computation. Although the proposed method requires slightly more processing time, it achieves superior reverberation suppression performance.

The field data were collected in actual ocean environments with considerable interference, and only the precise location of one underwater target was recorded. Another two trajectories from ship targets are also detected, suggesting that the proposed method is applicable for multi-target detection, and the dynamic components extracted by the method are irrelevant to the number of targets.

If there are no targets entering the sonar monitoring area, the dynamic component contains reverberation fluctuations or other non-target interferences, such as marine creatures. These non-target signals may lead to false positives in some pings. Nonetheless, false positives seldom show evolutionary continuity in the spatial-time space. Thus, the existence of a target is jointly determined by its appearance across multiple pings. The WSTD method separates targets and interferences by exploiting their differences across multiple pings, regardless of the number of targets.

Previous methods have achieved reverberation reduction based on the observation matrix shown in Figure 1a. However, this approach fails to accurately capture variations in reverberation backgrounds across different directions, leading to imprecise estimation of the steady component of reverberation. In contrast, the proposed method performs low-rank and sparse matrix decomposition on the multi-ping beamforming output for each direction individually, effectively capturing background variations and accurately extracting target echoes, even under low SRR conditions. When the underwater acoustic environment remains relatively stable, the performance of the proposed method is comparable to that of previous methods. The primary advantage of the proposed method lies in its enhanced robustness in scenarios where reverberation background variations are pronounced. Furthermore, in practical monitoring tasks, it may not be necessary to monitor all directions. Therefore, the proposed method can be used to detect the presence of targets in specific directions.

## 6. Conclusions

In this paper, we analyzed the correlation of multi-ping beamforming outputs from different directions using the correlation coefficient. We found that these correlations can vary significantly due to the time-varying characteristics of the underwater acoustic channel, leading to a decrease in the reverberation suppression performance of the previous method. To address this issue, we proposed a method based on AccAltPro that decomposes the beamforming outputs from different directions into steady and dynamic components separately using parallel computation. An improved WSTD method with adaptive threshold selection is then employed to further extract target echoes from the dynamic component. Field experiment data collected in Sanya, China, demonstrate the effectiveness of the proposed method. Compared with the LRMA method, the ISLR of the proposed method increases by an average of 0.87 dB, and it is increased by a factor of 1.04.

The proposed method enhances the robustness of low-rank sparse decomposition algorithms in scenarios with significant variations in reverberation background, enabling the detection of target echoes even in low signal-to-reverberation ratios (SRRs). Future research will focus on the generalization performance of the algorithm, testing its reverberation suppression capabilities in different maritime environments.

## Data Availability

Dataset available on request from the authors.
